# Exogenous Liposomal Ceramide-C6 Ameliorates Lipidomic Profile, Energy Homeostasis, and Anti-Oxidant Systems in NASH

**DOI:** 10.3390/cells9051237

**Published:** 2020-05-16

**Authors:** Francesca Zanieri, Ana Levi, David Montefusco, Lisa Longato, Francesco De Chiara, Luca Frenguelli, Sara Omenetti, Fausto Andreola, Tu Vinh Luong, Veronica Massey, Juan Caballeria, Constantino Fondevila, Sriram S Shanmugavelandy, Todd Fox, Giuseppe Mazza, Josepmaria Argemi, Ramon Bataller, Lauren Ashley Cowart, Mark Kester, Massimo Pinzani, Krista Rombouts

**Affiliations:** 1 Department of Experimental and Clinical Medicine and Center of Excellence “DENOthe”, University of Florence, 50134 Florence, Italy; francescazanieri@yahoo.it (F.Z.); sara.omenetti@gmail.com (S.O.); m.pinzani@ucl.ac.uk (M.P.); 2 University College London UCL, Institute for Liver & Digestive Health, Royal Free, London NW3 2PF, UK; ani.levi@outlook.com (A.L.); lisa.longato@gmail.com (L.L.); francesco.chiara@ucl.ac.uk (F.D.C.); luca.frenguelli@ucl.ac.uk (L.F.); f.andreola@ucl.ac.uk (F.A.); tuvinh.luong@nhs.net (T.V.L.); giuseppe.mazza.12@ucl.ac.uk (G.M.); 3 Virginia Commonwealth University, Richmond, Virginia, VA 23219, USA; david.montefusco@vcuhealth.org (D.M.); lauren.cowart@vcuhealth.org (L.A.C.); 4 Royal Free Hospital, Department of Cellular Pathology, London NW3 2PF, UK; 5 Division of Hepatology and Gastroenterology, Departments of Medicine and Nutrition, University of North Carolina at Chapel Hill, Chapel Hill, NC 27599, USA; sheth.massey@gmail.com (V.M.); bataller@pitt.edu (R.B.); 6 Hepatology, Hospital Clínic, IDIBAPS, CIBERehd, 08036 Barcelona, Spain; caballer@clinic.cat; 7 Liver Transplant Unit, Department of Surgery, Hospital Clinic, University of Barcelona, 08036 Barcelona, Spain; cfonde@clinic.cat; 8 Penn State University College of Medicine, Department of Pharmacology, Hershey, PA 17033, USA; sriram.saravanan@gmail.com (S.S.S.); tef3b@virginia.edu (T.F.); mk5vq@virginia.edu (M.K.); 9 Department of Medicine, Pittsburgh Liver Research Center, University of Pittsburgh, Pittsburgh, PA 15261, USA; j.argemi@pitt.edu; 10 Hunter Holmes McGuire VA Medical Center, Richmond, VA 23219, USA; 11 Department of Pharmacology, University of Virginia School of Medicine, PO Box 800735, Charlottesville, VA 22908, USA; 12 Sheila Sherlock Liver Centre, Royal Free London NHS foundation trust, London NW3 2PF, UK

**Keywords:** non-alcoholic steatohepatitis (NASH), human hepatic stellate cells (hHSC), liposomes, ceramides, adenosine monophosphate-activated kinase (AMPK), nuclear factor-erythroid 2-related factor 2 (Nfe2l2/NRF2), inflammation, apoptosis, phosphatidylcholine (PC), diacylglycerol (DG), lipidomics, methionine-choline deficient diet (MCD)

## Abstract

In non-alcoholic steatohepatitis (NASH), many lines of investigation have reported a dysregulation in lipid homeostasis, leading to intrahepatic lipid accumulation. Recently, the role of dysfunctional sphingolipid metabolism has also been proposed. Human and animal models of NASH have been associated with elevated levels of long chain ceramides and pro-apoptotic sphingolipid metabolites, implicated in regulating fatty acid oxidation and inflammation. Importantly, inhibition of de novo ceramide biosynthesis or knock-down of ceramide synthases reverse some of the pathology of NASH. In contrast, cell permeable, short chain ceramides have shown anti-inflammatory actions in multiple models of inflammatory disease. Here, we investigated non-apoptotic doses of a liposome containing short chain C6-Ceramide (Lip-C6) administered to human hepatic stellate cells (hHSC), a key effector of hepatic fibrogenesis, and an animal model characterized by inflammation and elevated liver fat content. On the basis of the results from unbiased liver transcriptomic studies from non-alcoholic fatty liver disease patients, we chose to focus on adenosine monophosphate activated kinase (AMPK) and nuclear factor-erythroid 2-related factor (Nrf2) signaling pathways, which showed an abnormal profile. Lip-C6 administration inhibited hHSC proliferation while improving anti-oxidant protection and energy homeostasis, as indicated by upregulation of Nrf2, activation of AMPK and an increase in ATP. To confirm these in vitro data, we investigated the effect of a single tail-vein injection of Lip-C6 in the methionine-choline deficient (MCD) diet mouse model. Lip-C6, but not control liposomes, upregulated phospho-AMPK, without inducing liver toxicity, apoptosis, or exacerbating inflammatory signaling pathways. Alluding to mechanism, mass spectrometry lipidomics showed that Lip-C6-treatment reversed the imbalance in hepatic phosphatidylcholines and diacylglycerides species induced by the MCD-fed diet. These results reveal that short-term Lip-C6 administration reverses energy/metabolic depletion and increases protective anti-oxidant signaling pathways, possibly by restoring homeostatic lipid function in a model of liver inflammation with fat accumulation.

## 1. Introduction

Nonalcoholic fatty liver disease (NAFLD) represents a major health issue worldwide, with evolution into non-alcoholic steatohepatitis (NASH) in 10% of cases, and possible further progression to liver cirrhosis and hepatocellular carcinoma (HCC) [1,2,3]. Consequently, major efforts are currently dedicated to discovering targetable pathogenic mechanisms and designing strategies able to reduce or arrest disease progression [4,5].

Studies in patients with NASH and animal models have provided evidence for an altered regulation of lipid metabolism in the progression from NAFLD to NASH [6,7]. In particular, human lipidomic/metabolomic studies have highlighted a significant increase of sphingolipid metabolites, including ceramides, in addition to elevations in tri- and di-glycerides [8,9].

Ceramides constitute a family of sphingolipids that consist of sphingosine covalently linked to a fatty acid, which are generated through de novo synthesis from serine and palmitate, sphingomyelin hydrolysis, or by sphingosine recycling from sphingolipids in the endosomes. The pathways leading to the synthesis of ceramides have shown abnormal profiles in NASH [10,11]. In addition, NASH phenotypes are mitigated by generic inhibitors of de novo ceramide metabolism [12,13], or exacerbated by over-expression of ceramide synthases [14,15]. Regardless, the overall biological actions of ceramides in the development and progression of NASH are only partially defined [11,16,17].

In broader terms, ceramide and its metabolites have profound effects on cellular metabolism and energy homeostasis, leading to a shift from anabolic to catabolic pathways [18,19,20]. Along these lines, multiple ceramide species have been shown to contribute to ectopic lipotoxicity and may interfere with cellular signaling pathways, thus promoting insulin resistance and type 2 diabetes [21,22]. Ceramides have also been shown to exert a negative impact on the regulation of energy homeostasis, including the inhibition of the energy-sensor adenosine monophosphate activated kinase (AMPK) phosphorylation [23] and transcription factors such as nuclear factor-erythroid 2-related factor 2 Nrf2 (Nfe2l2) [24,25]. In addition, AMPK phosphorylates Nrf2 (Ser550) [25,26,27], which regulates transcriptional induction of antioxidant response element (ARE)-containing genes encoding antioxidant enzymes, electrophile-conjugating enzymes, ubiquitin/proteasomes, and chaperone/heat-shock proteins in response to oxidative stress [27]. Therefore, ceramides may also reduce the natural antioxidant response and favor lipid peroxidation and progression of NASH.

On the other hand, evidence suggests that the overall effects of ceramides may be dependent on the dosing regimen, specific chain-lengths, and inherent hydrophobicity and impermeability [14]. It has been demonstrated that short-chain C6-ceramide can actually exert anti-inflammatory and anti-lipogenesis effects [28,29], and we are proposing Lip-C6, a non-toxic hydrophilic delivery liposome vehicle containing ceramide-C6, specifically designed for systemic administration [30,31,32]. Remarkably, cell permeable ceramides can increase nuclear translocation and DNA binding of Nrf2, as well as c-jun, to control ARE-mediated transcriptional activity [33,34]. These ceramides also increase the interaction between Nrf2 and c-jun, leading to up-regulation of antioxidant enzymes and resultant amelioration of oxidative stress in astrocyte models [35]. 

In this study, we firstly show a perturbation in Nrf2 and AMPK pathway genes in the liver tissue of NASH patients. Further, we investigated the effects of non-apoptotic doses of the cell permeable ceramide Lip-C6 on AMPK-and Nrf2-dependent oxidative stress in an animal model recapitulating hepatic fat accumulation and tissue inflammation typical of NASH. Finally, the effects of Lip-C6 found in vivo were also investigated in primary human hepatic stellate cells, in key cells in liver fibrosis, and in the progression from NAFLD to NASH.

## 2. Materials and Methods

### 2.1. Reagents

All reagents used in this study were from Sigma Aldrich unless otherwise mentioned. The list of antibodies and qRT-PCR assays-on demand utilized are shown in Supplementary Tables S1 and S2, respectively.

### 2.2. RNA Sequencing of Human Tissue NAFLD

For human RNAseq studies, human liver samples were obtained from the Human Biorepository Core from the NIH-funded international InTeam consortium (7U01AA021908-05) as previously described [36]. All patients gave written informed consent and the research protocols were approved by the local Ethics Committees and by the central Institutional Review Board of the University of North Carolina at Chapel Hill. For the present study, we compared non-diseased normal human livers (N = 10) with NAFLD patients according to Kleiner’s Criteria and without alcohol abuse (N = 9) (Supplementary Table S3). Patients with malignancies were excluded from the study.

RNA extraction, sequencing, and bioinformatic analysis: RNA extraction and sequencing was performed as indicated previously [36]. Total RNA from flash-frozen liver tissue was extracted by phenol/chloroform separation (TRIzol, Thermoscientific, Waltham, MA, USA). RNA purity and quality were assessed by automated electrophoresis (Bioanalyzer, Agilent, Santa Clara, CA, USA) and sequenced using Illumina HiSeq2000 platform. Libraries were built using TruSeq Stranded Total RNA Ribo-Zero GOLD (Illumina, San Diego, California, USA). Sequencing was paired end (2 × 100 bp) and multiplexed. Ninety-four paired-end sequenced samples obtained an average of 36.9 million total reads with 32.5 million (88%) mapped to GRCh37/hg19 human reference. Short read alignment was performed using STAR alignment algorithm with default parameters. To quantify expression from transcriptome mappings we employed RSEM.

### 2.3. Preparation of Nanoliposomal C6-Ceramide

Briefly, 12% pegylated nanoliposomes (80 ± 15 nm in size) that contain 30 mol% ceramide were prepared as previously described with lipids 1,2-distearoyl-sn-glycero-3- phosphocholine, 1,2-dioleoyl-sn-glycero-3-phosphoethanolamine, N-hexanoyl-d-erythro-sphingosine (C6-ceramide), 1,2-distearoyl-sn-glycero-3-phosphoethanolamine-N-[methoxy polyethylene glycol-2000], and N-octanoylsphingosine-1-[succinyl(methoxy polyethylene glycol-750)] (PEG(750)-C8) combined in chloroform at a molar ratio of 3.75:1.75:3:0.75:0.75 [37]. Combined lipids were dried under C6-nitrogen gas and resuspended in 0.9% sterile NaCl at 60 °C. Following rehydration, the resulting solution was sonicated for 5 min, followed by extrusion through a 100 nm polycarbonate membrane using the Avanti Mini Extruder (Avanti Polar Lipids, Alabaster, Alabama, USA). Control ghost liposomes (Lip-G) were prepared in a similar manner, excluding N-hexanoyl-d-erythro-sphingosine (C6). Several Quality Assurance and Quality Control (QA/QC) parameters were evaluated after preparation of nanoliposomes that were formulated within the size range of 85 nM–90 nM, as measured by dynamic light scattering. Zeta potentials of the nanoliposomes were measured and were between –10 mV and –15 mV. For the MCD diet, each animal was given a single tail vein injection of 100 µl of a 25 mg/mL solution of liposomes containing ceramide C6 (Lip-C6) or ghost (Lip-G).

### 2.4. Animal Experiments

Methionine choline deficient diet (MCD): Male BALB/c cN SPF mice, six weeks old, weighing between 20 and 25 g, were purchased from Charles River Laboratories (Calco, Italy). All animals were housed five per cage and kept under a controlled temperature of 22 ± 2 °C, 50–60% relative humidity, and 12 h light/dark cycles. Injection of Lip-C6 and culling of animals were performed in the morning. Mice had free access to food and water ad libitum. One week after arrival, mice were subdivided and fed either a diet deficient in methionine and choline (MCD diet) or the same diet supplemented with methionine and choline (control diet, CD). Diets were prepared by Dottori Piccioni Laboratories (Milan, Italy) [38] and stored at 4 °C until used. After nine weeks on different diets, mice were further subdivided to receive a single tail vein injection of either C6-ceramide containing liposomes (Lip-C6) or empty liposomes (ghost, Lip-G), resulting in six experimental groups (CD n = 5; CD–Lip-G n = 5; CD–Lip-C6 n = 5; MCD n = 5; MCD Lip-G n = 5; and MCD–Lip-C6 n = 10). One week after the tail vein injection, all mice were euthanized via exsanguination under anesthesia with an i.p. injection of 80 mg/kg 50% tiletamine hydrochloride and 50% zolazepam hydrochloride (Zoletil, Virbac, France). Body weight was recorded. Blood samples were centrifuged at 4500 rpm for 15 min at 4 °C to obtain serum that was kept at −20 °C until analyzed. Livers were rapidly dissected, weighed, snap-frozen in liquid nitrogen, and kept at −80 °C for further analysis. A portion of the liver was immediately fixed in formalin for histological analyses. Experimental protocols were conducted according to established international guidelines (Guide for the Care and Use of Laboratory Animals, NIH publication No. 86-23) and after approval by the University of Florence and Italian National Regulatory Authorities.

### 2.5. Serum Aminotransferase Levels

Serum alanine-aminotransferase (ALT) and aspartate aminotransferase (AST) activities were determined using a commercially available kit and Reflotron (Roche Diagnostic, Milan, Italy), as previously described [38].

### 2.6. Liver Histology

A portion of liver tissue was fixed by immersion in 10% buffered formalin (pH 7.4) for 24 h. The fixed tissue was dehydrated in graded ethanol, paraffin-embedded, and sectioned at a thickness of 4 µm. Hematoxylin–eosin (H&E) and Sirius Red staining were performed as previously described [39]. Images were captured with an Axiocam IcC5 using Zeiss Axiovision (version 4.8.2). Liver histology was evaluated according to the NASH CRN scoring system [40] by an experienced hepato-pathologist (T.V.L.) blinded to the type of treatment received by the animals. Four histological variables commonly described in NASH were analyzed on the H&E stained sections: (1) the presence of predominantly macro-vesicular or large droplet steatosis, graded 0–3 based on percent of hepatocytes in the biopsy involved; (2) lobular inflammation, graded 0–3 based on inflammatory foci per 20× with a 20× ocular; (3) hepatocellular ballooning, graded 0–3 based on numbers of ballooned hepatocytes (none = 0, a few = 1 and many = 3); and (4) apoptotic bodies, counted per 20× and graded 0–3, as in lobular inflammation. Fibrosis assessment was based on the use of Sirius Red stained sections and evaluated according to the NASH CRN fibrosis staging system [40].

### 2.7. Human Hepatic Stellate Cell Isolation and Culture

Primary human hepatic stellate cells (hHSCs) were isolated from wedge sections of liver tissue, obtained from patients undergoing surgery in the Royal Free Hospital after giving informed consent (NC2015.020 (B-ERC-RF). Cells were isolated according to Mederacke et al. [41] with modifications for human liver [42]. Briefly, 10 g of total human liver tissue was digested with 0.01% Collagenase, 0.05% Pronase, and 0.001% DNase I without performing perfusion. The homogenate was filtered through a 100 µm cell strainer (BD Falcon) and the flow-through was centrifuged at 50× *g* for 2 min at 4 °C. After washing the supernatant, gradient centrifugation was performed at 1400× *g* for 17 min at 4 °C using an 11.5% Optiprep gradient. Finally, the interface was collected and washed. Purity of hHSC was established by detection of CD140b (PDGFRβ), CD29 (Integrin β1), and Cytoglobin B (CYGB).

The obtained hHSC were cultured in IMDM supplemented with 20% fetal bovine serum (FBS), glutamine, nonessential amino acids 1×, 1.0 mM sodium pyruvate, 1× antibiotic-antimycotic (all Life Technologies), referred to as complete HSC medium hereinafter. Experiments described in this study were performed on hHSC of at least three independent cell preparations/donors, between passage 3 and 8.

### 2.8. Cytotoxicity, Cell Proliferation and ATP Assays

Primary hHSC were seeded (density 26 × 10^3^/cm^2^) under basic serum-rich conditions for 24 h, followed by serum deprivation (serum free medium, SFM) for the next 24 h prior to liposomes exposure. Next, cells were treated for 24 h with a range of concentrations (100–12.5 µM) of either Lip-C6 or Lip-G. Cytotoxicity and cell proliferation were assessed by MTT/MTS test (Promega, Southampton, UK) and BrdU ELISA (Sigma Aldrich, Dorset, UK), respectively [43]. Moreover, the Lip-C6 inhibitory effect on cell proliferation was further quantified by employing ATP assay. Cells were seeded at a density of 10,000 cells/well/100 uL in a 96-well plate in culture medium and serum-starved for 24 h followed by incubation with 6.25 µM of Lip-C6 for up to 24 h. Cell lysis was induced using the CellTiter-Glo^®^ Reagent (Promega, Southampton, UK), according to the manufacturer’s specification. Luminescence was recorded and the intracellular ATP concentration was calculated from an ATP standard curve and normalized to hHSC proliferation, as measured by BrdU assay. All samples were assayed in quadruplicates and according to the manufacturer’s manual and as previously described [43].

For protein analyses, hHSC were exposed to non-cytotoxic doses of Lip-C6 and Lip-G (6.25 and 3.125 µM) for up to 24 h and total protein lysates were analyzed by Western blot, as described below.

In another set of experiments, liposome uptake was monitored in hHSC grown on glass chamber slides. After 24 h of incubation with the same non-cytotoxic doses of 6.25 µM rhodamine-labelled Lip-C6 and Lip-G liposomes, nuclei were counterstained with Hoechst 33342 (1 μM, final concentration) for 10 min, cells were then washed three times in 1× HBSS, and observed under a fluorescence microscope (AxioScopeA1, Carl Zeiss Ltd., Cambridge, UK).

### 2.9. RNA Isolation and Quantitative Real-Time PCR

Total RNA was extracted using Qiazol reagent and RNeasy Universal Mini Kit (Qiagen, Manchester, UK) and quality and quantity was assessed by Nanodrop 2000 Spectrophotometer. One µg of total RNA was reverse transcribed with random primers and MultiScribe RT enzyme (Applied Biosystems, Paisley, UK). Taqman^®^ gene expression assays were used (Supplementary Table S2) and the signal was acquired with Applied Biosystems 7500 Fast Real-Time PCR System (ThermoFisher Scientific, Paisley, UK). Data were expressed as 2-∆∆Ct and GAPDH served as endogenous control [44,45].

### 2.10. Protein Extraction and Western Blot Analysis 

Briefly, liver tissue and cells were lysed in RIPA buffer containing 20 mM/l Tris·HCl, pH 7.4, 150 mM NaCl, 5 mM EDTA, 1% Nonidet P-40, 1 mM Na3(VO)4, 1 mM/l PMSF, 1X proteinase inhibitor cocktail, and 0.05% aprotinin. Insoluble proteins were discarded by centrifugation at 10,000 rpm at 4 °C, and total proteins were measured (Pierce, Rockford, IL) and stored at −80 °C for further analysis. For immunoblot analysis, whole cell lysates (25–35 μg) were separated on SDS–PAGE, transferred to nitrocellulose, and immunoblotted as described previously. Equal loading was demonstrated by re-probing membranes with antibody against either β-actin, total actin, or vinculin [46].

### 2.11. Lipidomics

Liver lipids were extracted using methyl-tert-butyl ether as described by others [47]. Extracts were separated on a 2.1 mm x 10 cm C8 Ethylene Bridged Hybrid (BEH) column (Waters Milford, MA, USA) with 60:40 water/acetonitrile 10 mM ammonium acetate and 90:10 isopropranol/acetonitrile, 10 mM ammonium acetate as the mobile phases. Eluate was analyzed with an inline AB Sciex 5600 TripleTOF mass spectrometer (Sciex, Framingham, MA, USA) using information-dependent acquisition. Data were analyzed using Progenesis QI and SIMCA-P for multivariate analyses. Orthogonal partial least squares-discriminant analysis was performed to identify features that distinguished between the groups. A cut-off of VIP [2] score >3 was used for feature (lipid) identification.

### 2.12. Statistical Analysis

**Figure 1:**
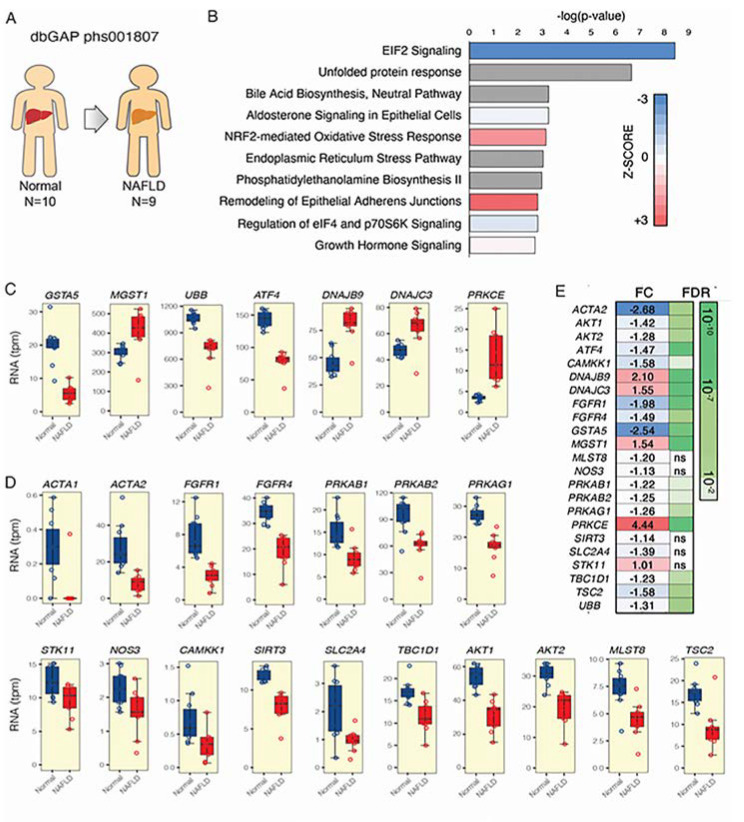
Adenosine monophosphate-activated kinase (AMPK) subunits and Nrf2 gene expression are significantly changed in nonalcoholic fatty liver disease (NAFLD) patients. RNA sequencing was performed on (**A**) normal (N = 10) and NAFLD derived liver tissue (N = 9 patients). (**B**) Summary of the functional enrichment analysis of the differentially expressed transcriptome using ingenuity pathway analysis. The significance of each gene set is indicated in the bar graph. The top functions belonging to the canonical pathway class are represented. The colors of the bars indicate the direction (blue meaning inhibition, and red activation) and the Z-score, which measures the magnitude (intensity of color) for the enrichment of a specific pathway. For some functions, no Z-score was calculated because of a lack of enough evidence regarding the specific gene functions or expression within a specific gene set (gray bars). The pathway “NRF2-mediated oxidative stress response” was significantly upregulated in patients with NAFLD. (**C**–**D**) Gene expression related to different Nrf2 and AMPK signaling pathways/mechanisms shown to be altered. (**E**) Representations of fold change (FC) and false discovery ratio (FDR) are shown for each gene investigated.

Body/liver weight, ALT/AST quantification, and cellular assays were analyzed with two-way analysis of variance (ANOVA) and Tukey’s multiple comparisons test using Prism software (Graph Pad, CA, USA). Semi-densitometry analysis for protein analysis was performed by employing Fiji ImageJ and GraphPad Prism was used for standard error (SE). Histograms represent averages ± SE of two samples of each of the conditions investigated. Statistical analysis for RNA sequencing: data are shown as box plots, indicating the median, the inter-quartile range, the maximum and minimum values, and occasionally the outliers. The comparison between normal and NAFLD RNA counts was made using a differential expression analysis and fitting a linear model using limma package. The *p*-value was corrected with the Benjamini–Hochberg method to calculate the false discovery rate for each gene in Figure 1, panels C, D, and E. qPCR data were analyzed using unpaired T test and the mean value of the controls was set to 1. Data were analyzed with Prism software (Graph Pad, CA, USA). Lipidomics data were analyzed using Progenesis QI and SIMCA-P for multivariate analyses. Orthogonal partial least squares-discriminant analysis was performed to identify features that distinguished between the groups. A cut-off of VIP [2] score >3 was used for feature (lipid) identification.

## 3. Results

### 3.1. AMPK and Nrf2 Signaling Pathways are Significantly Affected in NAFLD Patients

Unbiased transcriptomic and ingenuity pathway analyses (IPA) revealed that the Nrf2-mediated oxidative stress response network was upregulated in liver biopsies from NAFLD patients. Gene products associated with Nrf2-mediated transcription–oxidative stress response were upregulated (Figure 1A,B). Of interest, two Nrf2 pathway genes, implicated in the cellular stress-induced antioxidant response, and more specifically MGST1, which regulates inflammatory eicosanoid responses, was upregulated, and GSTA5, which catalyzes glutathione conjugation, was downregulated in NAFLD [48] (Figure 1C,E). Genes belonging to the unfolded protein response (ATF-4) [49] and ubiquitin B (UBB), which is necessary to fulfill the protein ubiquitination and one of the most important proteins in post-translational modifications, was significantly downregulated in NAFLD patients. The endoplasmic reticulum stress-related genes (DNAJB9, DNAJC3) and those involved in insulin resistance (PRKCε) [50] were all strongly upregulated in NAFLD patients.

No AMPK-related pathway was significantly enriched in NAFLD patients. We thus unbiasedly generated a list of AMPK related genes by means of STRING analysis (http://string-db.org/) [51] and UCSC Genome Browser data mining tool goldenPath (https://genome.ucsc.edu/goldenPath) [52] (Supplementary Figure S1A,B), and evaluated this particular set of AMPK-related genes in the RNA-seq of patients with NAFLD (Figure 1D,E). Besides the downregulation of several AMPK subunits (PRKAB1, PRKAB2, PRKAG1) [26] in NAFLD patients, most strikingly, a perturbation was found in many AMPK pathway genes such as those affecting the insulin/glucose homeostasis and downstream regulators (TBC1D1, SLC2A4/GLUT4, AKT1/2) [53], those genes involved in the regulation of lipid metabolism and activation/phosphorylation of AMPK (Sirt3) [54], and TSC2 regulating AMPK activation [55] displayed significant downregulation in NAFLD patients. Overall, these results suggested a dysregulated expression of several Nrf2 and AMPK pathway genes in NAFLD patients’ tissue indicating that both the endogenous anti-oxidant/detoxifying system and the AMPK-related energy homeostasis are altered in NAFLD patients and could represent possible targets for treatment.

### 3.2. Lip-C6 Affects Proliferation, and Promotes Phosphorylation of AMPK and the Endogenous Anti-Oxidant System in Human Primary HSC

**Figure 2:**
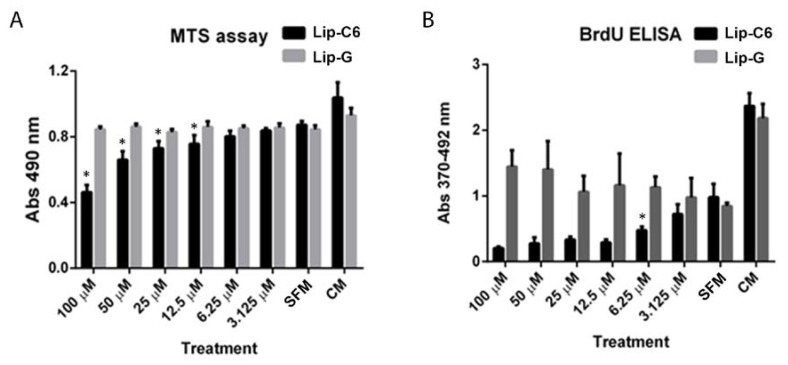

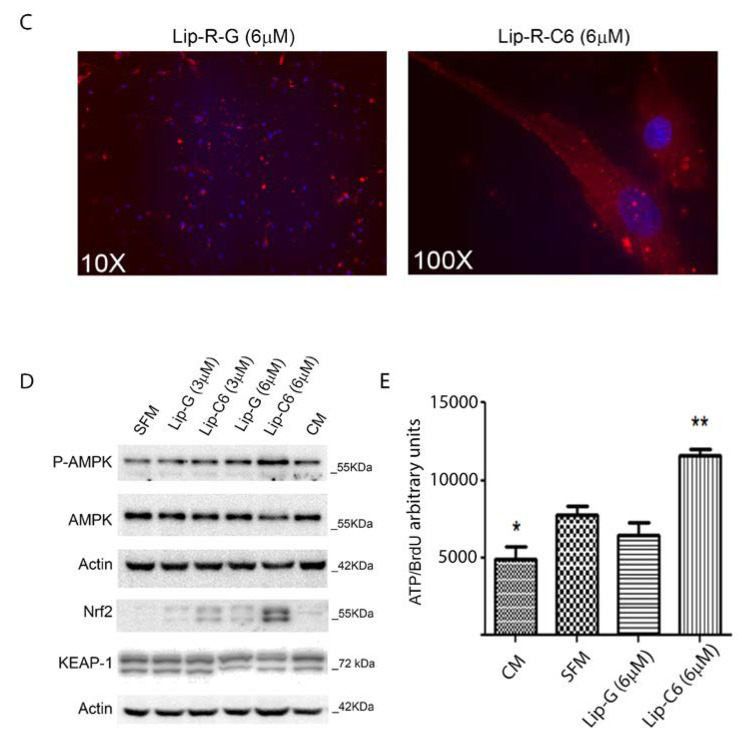
Liposomes containing short-chain ceramide C6 affect AMPK activation and proliferation, and promote the endogenous anti-oxidant system in primary human HSCs. Primary hHSCs were exposed to various concentrations (100–3.125 µM) of Lip-C6 or Lip-G for up to 24 h. (**A**) Higher doses of Lip-C6-treatment induced cytotoxicity (100–12.5 µM) (n = 4 per condition) (* *p* < 0.05), whereas (**B**) 6.25 µM inhibited hHSC proliferation (* *p* < 0.05) compared with serum free medium (SFM) (n = 4 per condition). (**C**) Representative images of liposomal uptake, which were evaluated by employing immunofluorescence and showed that liposomes containing ceramide-C6 with rhodamine (6.25 µM) were taken up as early as two hours after exposure. (**D**) Primary hHSCs were exposed to 6.25 and 3.125 µM of Lip-C6 for up to 24 h. Representative protein analysis showed an increase in phosphorylation of AMPK and upregulation of Nrf2 protein expression (n = 1 of 3 independent experiments). (**E**) Changes in ATP were observed when cells were exposed to 6.25 µM of Lip-C6 for up to 24 h (n = 4 per condition, * *p* < 0.05, ** *p* < 0.005 compared with SFM), Complete Medium (CM).

As hepatic stellate cells (HSCs) are key cellular effectors in the progression from NAFLD to NASH and liver fibrosis, we investigated the effect of Lip-C6 treatment on energetic, metabolic, and anti-oxidant signaling pathways in cultures of primary human HSC (hHSC) in vitro. Cultured activated, serum-starved primary hHSCs were exposed to various concentrations (100 µM–3.125 µM) of Lip-C6 or Lip-G as control, for 24 h. Higher doses of Lip-C6-treatment induced cytotoxicity (100 µM–12.5 µM, Figure 2A), whereas 6.25 µM Lip-C6 significantly inhibited hHSC proliferation without inducing cytotoxicity (Figure 2B). Next, immunofluorescence was performed and showed that liposomes containing rhodamine-labelled Lip-C6 and ghost were taken up as early as two hours after treatment (Figure 2C). On the basis of these data, we utilized non-toxic doses of Lip-C6 to investigate alterations of energy homeostasis and endogenous anti-oxidant signaling pathways in hHSC. Cells were exposed to 6.25 and 3.125 µM of Lip-C6 for up to 24 h. Protein analysis showed an increase in activation/phosphorylation of AMPK and an upregulation in Nrf2 protein expression (Figure 2D). These data indicate that Lip-C6 inhibits proliferation in hHSC through activation of AMPK, which is possibly because of an increased production in ATP. Indeed, changes in ATP homeostasis were observed in cells exposed to 6.25 µM of Lip-C6 for up to 24 h and showed increased ATP production (* *p* < 0.05, ** *p* < 0.005 compared with SFM) (Figure 2E).

### 3.3. Liposomal Treatment with Ceramide-C6 in MCD-Induced Liver Steatosis

**Figure 3:**
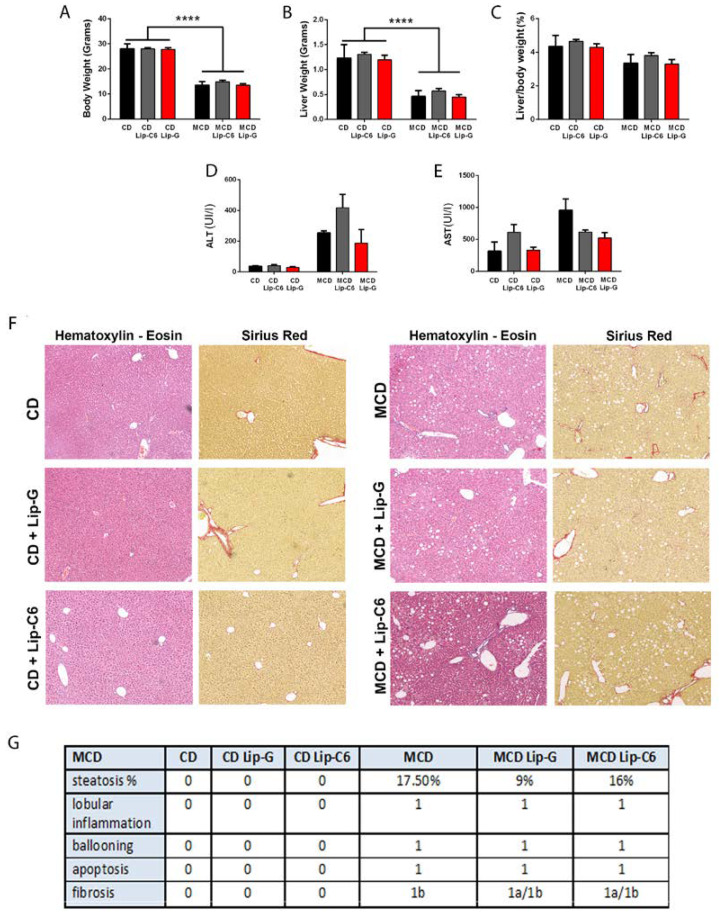
Liposomal treatment with ceramide-C6 in methionine-choline deficient (MCD)-induced liver steatosis. (**A**) Animals fed the MCD diet have significant loss of body weight regardless of treatment with Lip-C6 or Lip-G in comparison with the control diet (CD) group (**** *p* < 0.0001). (**B**) This coincided with a strong significant decrease in liver size in comparison with the control diet (CD) group (**** *p* < 0.0001) without (**C**) changes in liver/body weight ratio when comparing the control diet with MCD-fed mice. (**D**,**E**) No significant differences were observed between alanine transaminase (ALT) and aspartate aminotransferase (AST) levels of animals treated with Lip-C6 or Lip-G in comparison with their specific control condition. (**F**) Lip-C6 treatment did not exacerbate the MCD diet, as analyzed by hematoxylin and eosin and Sirius Red. (**G**) NASH CRN scoring system demonstrating changes occurring during Lip-G and Lip-C6 treatment in MCD-fed mice (CD n = 5; CD–Lip-G n = 5; CD–Lip-C6 n = 5; MCD n = 5; MCD Lip-G n = 5; and MCD–Lip-C6 n = 9).

In this set of experiments, the possible beneficial effects of Lip-C6 treatment were analyzed in an in vivo model of fatty liver associated with inflammation and fibrosis, recapitulating some aspects of NASH. Animals fed the MCD diet had significant body weight loss and decrease in liver size regardless of the treatment with Lip-C6 or Lip-G in comparison with the control diet group (Figure 3A,B **** *p* < 0.001). This coincided with no significant changes in the liver/body weight ratio (Figure 3C). Administration of Lip-C6 or Lip-G did not alter the MCD-induced increase in ALT and AST levels (Figure 3D,E). Histopathology data showed that MCD diet caused an accumulation of fat in hepatocytes, induced infiltration of inflammatory cells as stained with H&E, and collagen accumulation as stained with Sirius Red staining (Figure 3F). The NASH CRN scoring demonstrated that MCD-fed mice showed the presence of steatosis, mild lobular inflammation with minor hepatocellular ballooning, and a fibrosis scoring of 1a (mild/delicate zone 3 perisinusoidal fibrosis) and 1b (moderate/dense zone 3 perisinusoidal fibrosis), in comparison with the control diet (Figure 3G).

### 3.4. Lip-C6 Treatment in MCD Increases AMPK Phosphorylation/Activation, without Inducing Apoptosis in the MCD-Diet Model

**Figure 4:**
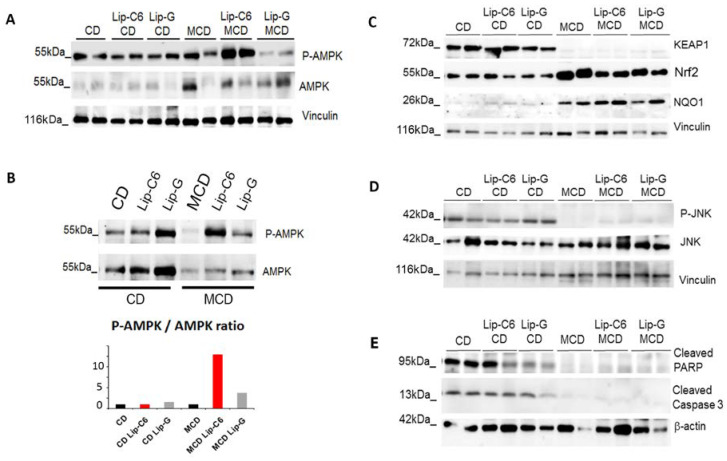
Liposome containing short-chain ceramide C6 treatment in MCD diet increases AMPK activation/phosphorylation and enhances the endogenous anti-oxidative stress signaling pathway without inducing apoptosis. (**A**) Representative Western blot analysis demonstrated an upregulation in activation/phosphorylation of AMPK by Lip-C6 treatment in MCD-fed mice in comparison with MCD-fed mice (n = 2 for each condition). (**B**) Inter-variability was assessed by protein analysis of pooled samples of each condition and densitometry scanning. MCD-fed mice have reduced AMPK protein levels in comparison with control diet-fed mice. Absolute levels of phosphorylated AMPK (P-AMPK) were highly induced in MCD-fed mice treated with Lip-C6 relative to MCD-fed mice and MCD-fed Lip-G treated mice. (**C**) Representative Western blot analysis demonstrated that Keap-1 protein expression was absent in MCD-fed mice, whereas Nrf2 and NQO1 protein expression were upregulated in MCD-fed mice in comparison with CD-fed mice (n = 2 for each condition). (**D**) Lip-C6 treatment in MCD-fed mice did not induce apoptosis by JNK activation/phosphorylation. (**E**) Cleaved poly (ADP-ribose) polymerase (PARP) and cleaved Caspase 3 protein expression showed to be absent in MCD-fed mice with no changes observed in Lip-C6 treated mice (n = 2 for each condition).

The effect of Lip-C6 treatment on AMPK and Nrf2 was investigated and showed that Lip-C6 treatment induced a strong phosphorylation of AMPK in MCD-fed mice (Figure 4A, Supplementary Figure S3A). To exclude intra-variability within each group, samples belonging to each specific condition were pooled and protein analysis was performed. Densitometric analysis showed that MCD-fed mice had reduced AMPK protein levels in comparison with control diet-fed mice. Absolute levels of phosphorylated AMPK (P-AMPK), a marker of AMPK activation, were highly induced in MCD-Lip-C6 treated mice relative to both MCD-fed and MCD- Lip-G treated mice (Figure 4B). In this study, both Nrf2 and NQO1 protein expression were upregulated in MCD-fed mice in comparison with CD-fed mice, whereas Keap-1 protein expression was absent in MCD-fed mice (Figure 4C). Of interest, Lip-C6 did not alter Keap-1 or NQO1 levels in the control or MCD diet groups, but did slightly reduce Nrf2 protein expression induced by the MCD diet (Figure 4C, Supplementary Figure S3B). To see if any of these effects coincided with apoptosis under any of the conditions analysed and, more specifically, whether Lip-C6 injection coincided with cell death, several apoptotic markers and signalling pathways were investigated. The phosphorylation of the pro-apoptotic c-Jun N-terminal kinase (JNK) was investigated, as an increase in endogenous oxidative stress can induce JNK activation. Our data showed that JNK phosphorylation was absent in all MCD-fed mice in comparison with the control diet (Figure 4D). Further, cleaved poly (ADP-ribose) polymerase (PARP) and cleaved caspase 3 protein expression were not observed in MCD-fed mice, with or without Lip-C6 treatment (Figure 4E, Supplementary Figure S3C,D). Moreover, phosphorylation of p62, which is regulated by the mRNA stabilizing protein HuR/ELAV1 and is known as an apoptotic marker, was not observed in MCD-fed mice, with or without Lip-C6 treatment, indicating that the Lip-C6 treatment does not induce Keap1/p62 related apoptosis in our model (Supplementary Figure S2A).

### 3.5. Lip-C6 Does Not Alter the Pro-Inflammatory Response in MCD-Fed Mice

**Figure 5:**
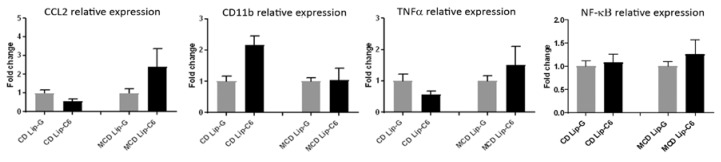
Lip-C6 does not exacerbate the pro-inflammatory response in MCD-fed mice. qRT-PCR showed that the Lip-C6 treatment in MCD-fed mice did not induce significant changes in mRNA expression of pro-inflammatory key genes versus internal control diet (CD) (CD n = 2, CD-G n = 5, CD-C6 n = 5, MCD n = 2, MCD-G n = 5, and MCD-C6 n = 8, mean value of controls was set to 1).

In reason of the protective anti-oxidant Nrf2 and energetic AMPK signaling networks induced by Lip-C6 in the in vivo model, the pro-inflammatory mechanisms potentially affected by these pathways were analyzed. Quantitative RT-PCR showed that Lip-C6 treatment did not significantly affect gene expression in MCD-fed mice (Figure 5), as no significant changes were observed in CC chemokine ligand 2 (CCL2), CD11b, tumour necrosis factor α (TNFα), and nuclear factor kappa B (NF-kB) mRNA transcripts from control and MCD diets in the presence or absence of Lip-C6.

### 3.6. Lip-C6 Treatment Restores Specific Phosphatidylcholines and Diacylglycerides in MCD-Fed Mice

**Figure 6:**
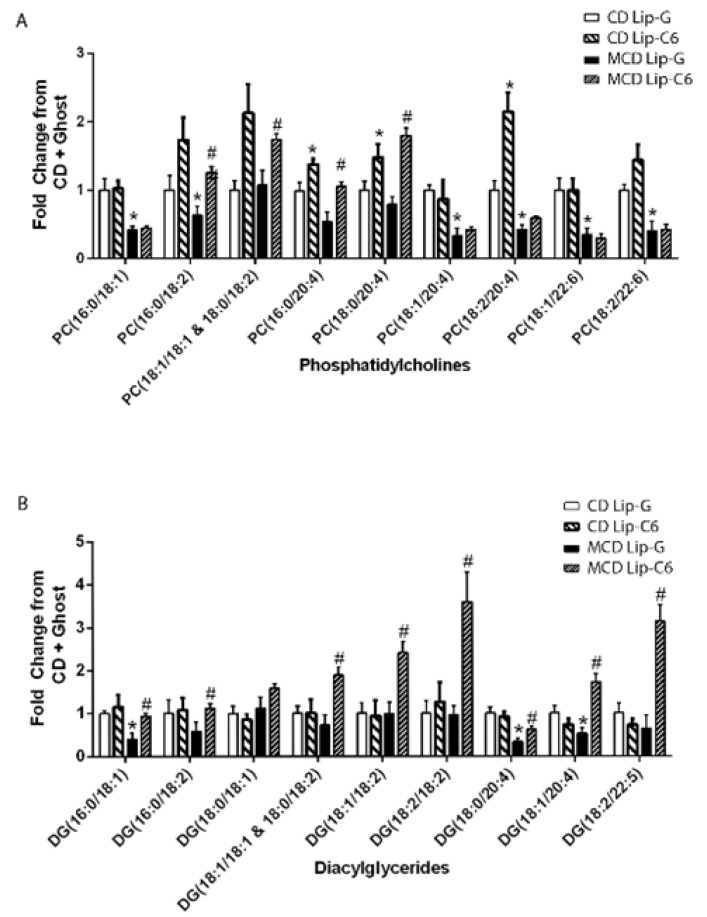
Lip-C6 treatment restores specific phosphatidylcholines (PC) and diacylglycerides (DC) in MCD-fed mice. Animals received MCD or CD diet for nine weeks and were further subdivided and administered a single tail vein injection of Lip-C6 or Lip-G. One week after treatment, all mice were euthanized. Lipids from liver samples were extracted and untargeted LC-MS/MS lipidomics approach was performed. (**A**) Major changes were observed in the lipid classes of phosphatidylcholine (PC) and (**B**) diacylglycerol classes (DG) when MCD-fed mice treated with Lip-C6 were compared with MCD-fed mice treated with Lip-G. No changes were detected for short-chain DG and TG. * *p* < 0.05 CMCD + Lip-G (n = 5) vs. MCD + Lip-G (n = 5), CMCD + Lip-G vs. CMCD + Lip-C6 (n = 8) # *p* < 0.05 MCD + Lip-G vs. MCD + Lip-C6 (n = 9).

It is well established that the MCD diet significantly reduces phosphatidylcholine (PC) pools and decreases PC/phosphatidylethanolamine (PE) ratio, as well as diacylglycerol (DG) classes, which are critical to maintain membrane integrity [56,57]. To understand whether Lip-C6 treatment could resolve the dysregulated lipid metabolism in MCD-fed mice, we assessed lipidomics via an untargeted Liquid chromatography–mass spectrometry (LC-MS)-based approach, after a single tail vein injection of Lip-C6. Major changes were observed in the lipid classes of PC and DG. The MCD diet diminished the amount of multiple phosphatidylcholines, which included PC(16:0/18:1), PC(18:1/20:4), PC(18:2/20:4), PC(18:1/22:6), and PC(18:2/22:6) (Figure 6A), as well as a few diacylglycerides DG(16:0/18:1), DG(18:0/20:4), and DG(18:1/20:4) (Figure 6B). With the exception of PC(16:0/20:4), a single injection and short-term C6-ceramide treatment in MCD-fed mice did not rescue the decrease on these specific PCs. However, Lip-C6 treatment led to the elevation of other PCs that included PC (16:0/18:2), PC (18:1/18:1 and 18:0/18:2), PC (16:0/20:4), and PC (18:0/20:4). Lip-C6 also increased PC (18:0/20:4) and PC (18:2/20:4) in mice on the control diet (Figure 6A). Likewise, Lip-C6 treatment led to elevated levels of DG(16:0/18:1), DG(16:0/18:2), DG(18:1/18:1 and 18:0/18:2), DG(18:1/18:2), DG(18:2/18:2), DG(18:0/20:4), DG(18:1/20:4), and DG(18:2/22:5) in mice on the MCD diet, but not the control diet (Figure 6B). Overall, these data indicate that Lip-C6 treatment has a beneficial effect on the dysregulated lipid metabolism in MCD-fed mice.

## 4. Discussion

The available literature makes a strong case for ceramide accumulation as a contributor to NASH progression as elevations in ceramides are observed in patient samples and various models of NASH, and pharmacological and molecular strategies to reduce ceramide levels are associated with reduced NASH progression [11]. Despite this body of evidence, the actual effects of non-apoptotic doses of ceramide on the pathophysiological mechanisms of NASH, and, in particular, energy homeostasis, are still controversial.

For example, ultraviolet and H_2_O_2_ treatment generates ceramide, which activates AMPK [58], while short-chain ceramides inhibit Nrf2 activation [34], and knock out of acid ceramidase 3 augments C18:1-ceramide levels, which alleviates early inflammation, oxidative stress, and fibrosis in a mouse model of NASH [59]. It has also been suggested that ceramide-activated AMPK can limit nutrient-induced stress and autophagy [60]. Altogether, these evidences could suggest an unappreciated compensatory action of ceramides upon energy metabolism in NASH or, alternatively, that non-toxic sub lethal doses of exogenous ceramide may have unappreciated effects upon energy metabolism. Finally, it could be conceivable that ceramide metabolites, and not ceramide itself, have a role in NASH pathophysiology [61].

On the other hand, some studies have suggested that individual species of ceramide (C16 as compared with C24:1) are more inflammatory or apoptotic in NASH models [62]. On the basis of the available evidence, it seems that ceramide-C6, in contrast to physiological ceramides, is not as metabolically active, and exhibits unique improvement in energy metabolism surrogates, although the effects on AMPK and Nrf2 are unknown. Of note, short-chain C6-ceramide can exert anti-inflammatory and anti-lipogenesis effects [28,29]. For these reasons, we choose to evaluate the effects of Lip-C6, a non-toxic hydrophilic delivery liposome vehicle containing C6-ceramide, specifically designed for systemic administration [30,31]. Preclinical toxicology studies, including physiochemical characterization and PK/PD analyses, showed that this 90 nm sized, −8 mV, 15 molar percent PEG, 30 molar percent C6-ceramide nanoliposome is non-toxic, where ceramide is released from Lip-C6 by intra-bilayer movement [32,63,64]. While Lip-C6 has been demonstrated as an anti-neoplastic, pro-apoptotic, in vivo therapeutic agent by activating anti-tumor immune response of tissue-associated macrophages [65,66,67], the action of Lip-C6, at non-apoptotic doses, has not been investigated in models of NAFLD or NASH.

On the basis of the evidence of a dysregulation in Nrf2 and AMPK pathway genes in liver tissue of patients with NAFLD by employing transcriptomic and RNA sequence analyses provided by the present study, we evaluated the effects of Lip-C6 on the AMPK/Nrf2 pathway in primary human HSC, key cellular effectors in the progression from NAFLD to NASH. Our results indicate that incubation with Lip-C6 activated AMPK, induced ATP and Nrf2, without inducing cell death.

In addition, this possible beneficial effect of Lip-C6 was evaluated in an in vivo model of diet-induced steatohepatitis and liver fibrosis. Although the methionine and choline-deficient (MCD) mouse model does not recapitulate the pathophysiological background on NASH in a context of metabolic syndrome, the model provides insights on the association of steatosis, inflammation, and fibrosis within the liver tissue. In particular, the MCD diet promotes a dysregulation in anti-oxidant homeostasis, and thus this model can serve to investigate the putative effect of ceramide upon AMPK and Nrf2 signaling oxidative stress, inflammation, and fibrosis. Lip-C6 treatment significantly elevated AMPK, while reducing Nrf2 expression in mice treated with the MCD-diet.

Taken together, it is reasonable to speculate that elevations in Nrf2 transcriptional events are compensatory events to modulate the anti-oxidant stress responses in NASH, while the decrease in the energy-sensor AMPK expression, or activity, leads to a direct exacerbation of NASH pathology, which can be diminished by Lip-C6. Human NASH omics-studies have shown that a dysregulation in ceramides and a deficiency of both methionine and choline, essential precursors of hepatic phosphatidylcholine synthesis, provokes hepatic steatosis. The MCD diet used in this study is known to induce steatosis by choline deficiency [68,69,70,71,72,73]. In animals with the MCD diet, while liver injury and oxidative stress occur rapidly, inflammation and fibrosis develop only after prolonged feeding, typically longer than eight weeks, and the severity of the histological changes observed is dependent on the genetic background and gender of the mouse strain utilized. For example, Galastri et al. have identified that a lack of CCL2 in Balb/C MCD-fed mice reduces inflammation, oxidative stress, and fibrogenesis, but not in CCL2 deficient C57Bl/6 MCD-fed mice, indicating that the effects of CCL2 deficiency in mice and their response to MCD are markedly dependent on the mouse genetic background [74,75]. Moreover, single amino acid methionine deprivation triggers dramatic and specific transcriptional amino acid responses in genes such as Nrf2, thus indicating a link between methionine deficiency and the endogenous anti-oxidative stress response of Nrf2 [76]. This association was further confirmed in the MCD diet used in this study with increased Nrf2 protein levels in MCD-fed mice and a modest decrease upon Lip-C6 treatment.

Through an untargeted lipidomics analysis, the most significant features identified in mice on the MCD diet were characterized by a marked decrease of hepatic phosphatidylcholine and diacyglyceride levels. Surprisingly, a single Lip-C6 tail vein administration after nine weeks of MCD diet reversed these pathological decreases in PC and DG species. The change in profile, especially in 18:2 and 20:4 fatty acyl species, could reflect a global elevation or changes in omega-6 fatty acid uptake, metabolism, or oxidation. Of note, activated AMPK modulates fatty acid metabolism by controlling acetyl-CoA carboxylase, malonyl-CoA decarboxylase, and fatty acid synthase [77,78,79]. Alternatively, the enzyme sphingomyelin synthase, which generates diacylglycerides from phosphatidylcholine at the expense of forming sphingomyelin from ceramide may also be mediating the action of Lip-C6 to restore phosphatidylcholine levels [80].

Overall, the in vivo and in vitro data further support that AMPK and Nrf2 are potential Lip-C6 “drug-able” target genes/proteins to treat NAFLD/NASH. Furthermore, the link between restoration of physiological lipid metabolism and activation of protective energetic and anti-oxidant cascades by Lip-C6 treatment deserves further investigation.

## Supplementary Materials

The following are available online at https://www.mdpi.com/2073-4409/9/5/1237/s1, Figure S1: Overview of AMPK specific STRING analysis and UCSC Genome Browser datamining; Figure S2: Lip-C6 treatment in MCD-fed mice enhances the endogenous anti-oxidative stress-signaling pathway without inducing apoptosis; Figure S3: semi-quantitative densitometric values of all proteins were normalized for phosphorylated versus total protein and/or to β–actin or Tubulin; Table S1: Primary and secondary antibodies used in this study; Table S2: qRT-PCR Assays-on-demand used in this study (Applied Biosystems); Table S3. Baseline characteristics of patients and controls at the time of liver biopsy.

## Abbreviations

AMPK	Adenosine monophosphate-activated kinase
PRKAA1	AMPK-α1, catalytic subunit
PRKAA2	AMPK-α2, catalytic subunit
PRKAB1	AMPK-β1, non-catalytic subunit
PRKAB2	AMPK-β2, non-catalytic subunit
PRKAG1	AMPK-γ1, non-catalytic subunit
PRKAG2	AMPK-γ2, non-catalytic subunit
Nfe2l2/Nrf2	nuclear factor-erythroid 2-related factor 2
Keap1	Kelch ECH associating protein 1
NQO1	NAD(P)H quinone oxidoreductase 1
PARP	poly (ADP-ribose) polymerase
HuR	ELAVL1 RNA binding protein
ALT	alanine transaminase
AST	aspartate aminotransferase
CCL2	CC chemokine ligand 2
CD11B	Itgam, cluster of differentiation molecule 11B
GAPDH	glyceraldehyde-3-phosphate dehydrogenase
MCD diet	methionine/choline-deficient diet
NAFLD	non-alcoholic fatty liver disease
NASH	non-alcoholic steatohepatitis
ROS	reactive oxygen species
RT-QPCR	real-time quantitative PCR
NF-ĸB	nuclear factor kappa B
TNFα	tumour necrosis factor α
PC	phosphatidylcholine
DG	diacylglycerol
GSTA5	Glutathione S-Transferase Alpha 5
MGST1	Microsomal Glutathione S-Transferase 1
UBB	protein ubiquitination
ATF-4	Activating Transcription Factor 4
DNAJB9	DnaJ Heat Shock Protein Family (Hsp40) Member B9
DNAJC3	DnaJ Heat Shock Protein Family (Hsp40) Member C3
PRKCε	Protein Kinase C Epsilon
ACTA1	Actin Alpha 1, Skeletal Muscle
ACTA2	Actin Alpha 2, Smooth Muscle
FGFR1-R4	fibroblast growth factor family receptors 1 and 4
LKB1	(STK11) Serine/Threonine Kinase 11
NOS3	Nitric Oxide Synthase 3
CAMKK1	Calcium/Calmodulin Dependent Protein Kinase Kinase 1
SIRT3	Sirtuin 3
SLC2A4	GLUT4 Solute Carrier Family 2 Member 4
TBC1D1	TBC1 Domain Family Member 1
AKT1–2	AKT Serine/Threonine Kinase 1 and 2
MLST8	MTOR Associated Protein
TSC2	TSC Complex Subunit 2
