# Supplementary material for: Exogenous Liposomal Ceramide-C6 Ameliorates Lipidomic Profile, Energy Homeostasis, and Anti-Oxidant Systems in NASH
Source: Cells. 2020 May 16;9(5):1237. doi: 10.3390/cells9051237 (PMC7290333; doi:10.3390/cells9051237)
Supplement: Supplementary file 1 [file cells-09-01237-s001.pdf]

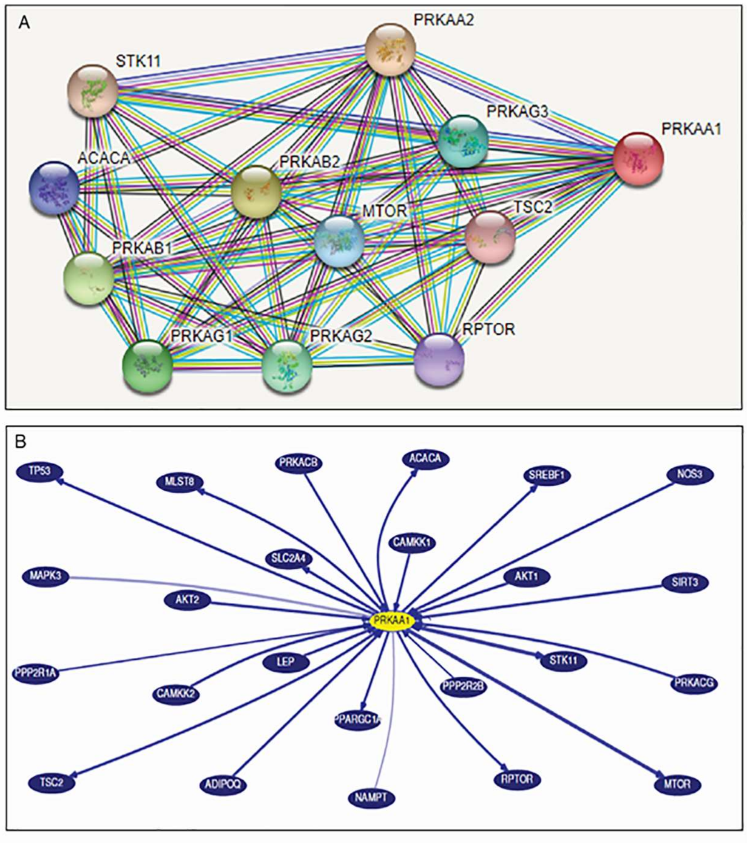

Figure S1: Overview of AMPK specific STRING analysis and UCSC Genome Browser datamining. (A) A list of AMPK closely related genes was generated by means of STRING analysis (http://string-db.org) and STRING analysis was performed, network edge evidence based and highest confidence (0.900) with no more than 10 interactors and (B) UCSC Genome Browser datamining (https://genome.ucsc.edu/goldenPath).

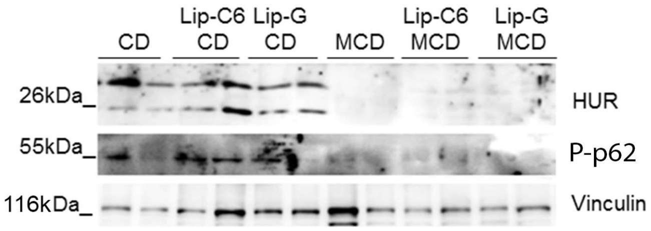

Figure S2. Lip-C6 treatment in MCD-fed mice enhances the endogenous anti-oxidative stress-signaling pathway without inducing apoptosis. Western blot analysis demonstrated that both expression of the p62 phosphorylation and the mRNA stabilizing protein HuR/ELAV1, known as apoptosis marker, were absent in MCD-fed mice with or without Lip-C6 treatment indicating that the Lip-C6 treatment does not induce KEAP1/p62 - related apoptosis (n=2 samples for each condition).

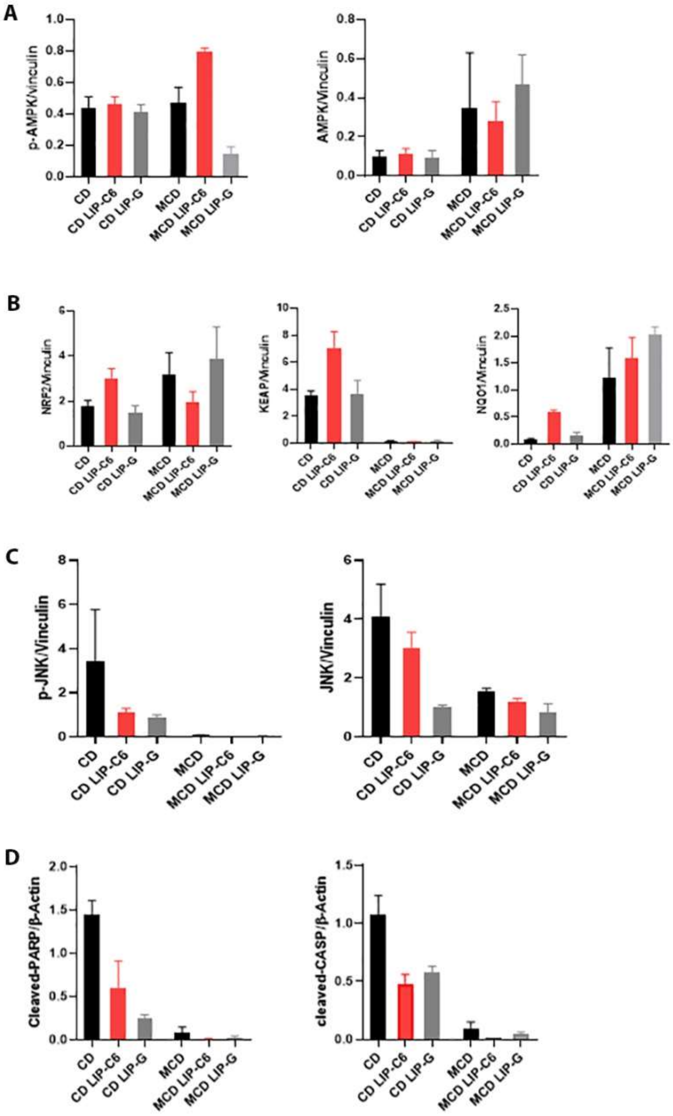

Figure S3. semi-quantitative densitometric values of all proteins were normalized for phosphorylated versus total protein and/or to β–actin or Tubulin. Densitometry analysis was performed by employing Fiji ImageJ and GraphPad Prism was used for Standard Error (SE). Graphs represent averages ± SE of 2 samples of each conditions investigated.

Table S1. Primary and secondary antibodies used in this study.

antibody	Catalogue number	Company
Cleaved PARP (D214) mouse specific	9544S	Cell Signaling technology
β-actin	A5441	Sigma-Aldrich
Cleaved Caspase 3 (D175)	9661S	Cell Signaling technology
Phospho-SAPK/JNK(Thr183/tyr185) (81E11)	4668S	Cell Signaling technology
SAPK/JNK(56G8)	9258S	Cell Signaling technology
Vinculin (clone hVIN-1 ascites fluid)	V9131	Sigma-Aldrich
Keap1	ab66620	Abcam
Actin (20-30)	A5060	Sigma-Aldrich
Nrf2	ab31163	Abcam
NQO1	ab2346	Abcam
HuR (H-280)	sc-20694	Santa Cruz Biotechnology
PhosphoSQSTM1/p62(Thr269/Ser272)	13121S	Cell Signaling technology
SQSTM1/p62	5114S	Cell Signaling technology
Phospho-AMPKα (Thr172) (40H9)	2535S	Cell Signaling technology

AMPKα	2532S	Cell Signaling technology
Donkey anti-goat IgG-HRP	sc-2020	Santa Cruz Biotechnology
Goat anti-mouse IgG-HRP	sc-2055	Santa Cruz Biotechnology
Goat anti-rabbit IgG-HRP	sc-2054	Santa Cruz Biotechnology
Goat anti-rabbit IgG pre-adsorbed (HRP)	GTX85336	GeneTex

Table S2. qRT-PCR Assays-on-demand used in this study (Applied Biosystems).

Gene	Abbreviation	Cat. N. (Company)
CC chemokine ligand 2	CCL2	Mm00441242_m1
cluster of differentiation molecule 11B	CD11B/Itgam	Mm00434455_m1
nuclear factor kappa B	NF-κB	Mm00476361_m1
tumor necrosis factor α	TNFα	Mm00443258_m1
Glyceraldehyde 3-phosphate dehydrogenase	GAPDH	Mm03302249_g1

Table S3. Baseline characteristics of patients and controls at the time of liver biopsy. WBC: white blood count; AST: aspartate aminotransferase; ALT: alanine aminotransferase; GGT: gamma-glutamyl transpeptidase; ALP: alkaline phosphatase.

	Normal livers n=10	NAFLD n=9
**Demographics**		
Age - median (IQR)	32(29-49)	49.5 (43-53)
Gender - male n (%)	7(70)	2(22.2)
**Lab parameters** - median (IQR)		
Hemoglobin g/dL	14.6(13.2-15.3)	14.3(12.4-14.9)
WBC x109/L	5.7(5.1-7.2)	8.13(6.9-10.1)
Platelets x109/L	237(217-267.5)	262(221.5-361.5)
AST (U/L)	21.5(19.2-25.8)	30(25-36.5)
ALT (U/L)	25(16.8-31.2)	40(31-49.5)
Bilirubin mg/dL	0.6(0.5-0.7)	0.6(0.4-0.88)
GGT (U/L)	17(14.0-23.2)	27(19-31)
ALP (U/L)	147(112.5-184.8)	182(170-195)
Albumin (g/dL)	4.6(4.4-4.6)	4.5(4.4-4.6)
Creatinine mg/dL	0.84(0.76-0.89)	1.05(0.84-1.1)
Sodium (mEq/L)	140(138.7-141.5)	140(136.5-142.5)
